# Real-World Marine Radar Datasets for Evaluating Target Tracking Methods

**DOI:** 10.3390/s21144641

**Published:** 2021-07-06

**Authors:** Jaya Shradha Fowdur, Marcus Baum, Frank Heymann

**Affiliations:** 1 Institute of Communications and Navigation, German Aerospace Centre (DLR), 17235 Neustrelitz, Germany; 2 Institute of Computer Science, University of Göttingen, 37077 Göttingen, Germany; marcus.baum@cs.uni-goettingen.de; 3 Institute for Solar-Terrestrial Physics, German Aerospace Centre (DLR), 17235 Neustrelitz, Germany; frank.heymann@dlr.de

**Keywords:** marine radar datasets, multiple target tracking, radar image processing, centroid-based tracking, data association

## Abstract

As autonomous navigation is being implemented in several areas including the maritime domain, the need for robust tracking is becoming more important for traffic situation awareness, assessment and monitoring. We present an online repository comprising three designated marine radar datasets from real-world measurement campaigns to be employed for target detection and tracking research purposes. The datasets have their respective reference positions on the basis of the Automatic Identification System (AIS). Together with the methods used for target detection and clustering, a novel baseline algorithm for an extended centroid-based multiple target tracking is introduced and explained. We compare the performance of our algorithm to its standard version on the datasets using the AIS references. The results obtained and some initial dataset specific analysis are presented. The datasets, under the German Aerospace Centre (DLR)’s terms and agreements, can be procured from the company website’s URL provided in the article.

## 1. Introduction

The need for a safe, collision-free and ecologically clean maritime navigation keeps getting more pronounced with increasing global trade demand [1,2,3]. Overseas trade is accompanied by concerns for safety and financial risks that can be potentially reduced by introducing vessel autonomy in maritime domains. This objective requires a continuous monitoring of vessels within a region of interest, also called observation region, where one desires to detect and track the vessels based on acquired sensor data. Automatic Identification System (AIS) is the technology used for providing the position of vessels, amongst other vessel and journey information, to the accuracy of a few metres, and it is mandatory for vessels above 300 gross tonnages to be equipped with the transponder [4,5,6]. It can be used either standalone or often coupled with an onboard radar. However, vessels equipped with AIS and related services such as the Global Navigation Satellite Systems (GNSS) are susceptible to malfunction due to jamming or spoofing attacks [6,7]. Therefore, the marine radar is still predominantly used as the standard navigation tool on boats and vessels today, being less prone to such vulnerabilities.

**Figure 1:**
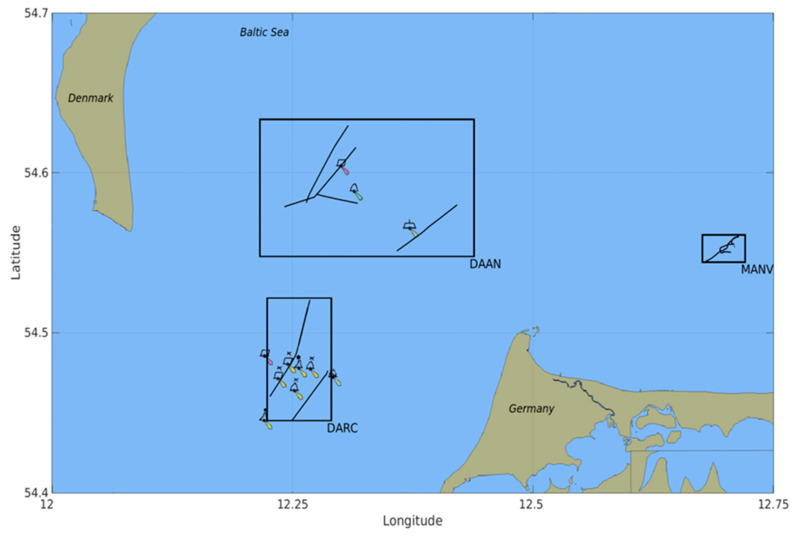
Observation regions during the measurement campaigns. The trajectories of each dataset are enclosed and labelled, in the Baltic Sea region. Note that only the aids to navigation present within the respective dataset’s observation region have been included in the plot.

The use of benchmark datasets has supported much development into various autonomous navigation applications through the recent years. For instance, the KITTI [8] and nuScenes [9] datasets are widely used datasets in the automotive fields so far. While the maritime research community has significantly focused on applications related to maritime surveillance, tracking and monitoring from real-world radar datasets [10,11,12,13,14,15,16], the datasets were unpublished for the most part. To the best of our knowledge, there are no real-world marine radar and AIS-based datasets that are accessible for research purposes so far. In this article, we hence present a repository containing a collection of real-world radar datasets covering common situations encountered from maritime routes that have been recorded from measurement campaigns carried out in the Baltic Sea by the German Aerospace Centre (DLR) (see Figure 1).

In addition to the datasets, we demonstrate the application of a tailored multi-target tracker on the same. Multiple target tracking (MTT) is the problem of jointly estimating the individual states of targets alongside the targets’ cardinality at each observation step [17]. Two main commonly used approaches to solve MTT problems are random finite set (RFS) [18,19] and traditional probabilistic data association (PDA). In the former, a finite set of individual target states is propagated as a density. The latter involves calculating state estimates based on probabilities of measurements being correctly assigned to a target [17,20]. A further point that can be considered in case of radars would be the sensor resolution, yielding the ability to track not only the kinematic properties of targets but as well as their extents (shape) since multiple measurements are highly likely to arise from the targets’ bodies. This problem is known as extended target tracking (ETT) [21]. In our conference papers [22,23], some initial work has been done using two of the datasets, where an ellipsoidal tracking method was proposed to estimate the orientation of dynamic vessels while keeping the extent parameters fixed. In this article, we however restrict the discussion to comparing our proposed extended centroid-based tracker with a basic centroid-target one in order to provide a baseline algorithm. We also present an improved automatic target detection approach that is faster, compared to the previous versions, and that can be included in a continuous processing chain from its raw form (i.e., images) to the targets state estimates.

There are three steps involved in the processing chain implemented in this work and are as summarised. In the first one, potential targets are extracted from radar images as point clouds. These point clouds are then subject to the k-means clustering approach [24,25] in the second step. Finally, the centroids thus obtained are fed to an adapted version of the joint probabilistic data association (JPDA) [20,26] filter to get the final multi-target estimates. The motivation behind adopting the last two steps is to process the set of point clouds in a more efficient manner, much in contrast to the previous methods in [22,23], where every point in the point cloud(s) had to be processed sequentially for the filter update at each observation step. Moreover, by the current method henceforth referred to as extended centroid-based JPDA (EC-JPDA), we are still able to somewhat retain each point cloud’s information in the form of its dispersion matrix. This concept was first brought by as a solution to the group tracking problem [26] and we propose applying it for tracking individual targets in an MTT perspective. In our case, the dispersion matrix is not only added to the innovation covariance at the gating stage, but it is also included in the calculation of association probabilities to account for the varying measurement spreads at different observation steps. The outcome of the tracker on each dataset is validated against the AIS reference positions and their results are presented and discussed. 

Our two-fold contribution is therefore as follows:

(1) We first introduce the DLR radar repository comprising three individual datasets along with the participating vessels’ reference positions as recorded from the AIS data.
(2) We then adapt the standard JPDA filter as a baseline algorithm for tracking centroids on the datasets in the repository to highlight the overall blockchain performance, compare the adapted version to the standard one and discuss the associated challenges.

The article is thusly organized: In Section 2, information about the datasets and data processing involved are described in detail. In the next one, we cover the target detection method, clustering and our filtering approach. The results are presented in Section 4, and we finally summarize the contents in Section 5.

## 2. Repository Description

**Figure 2:**
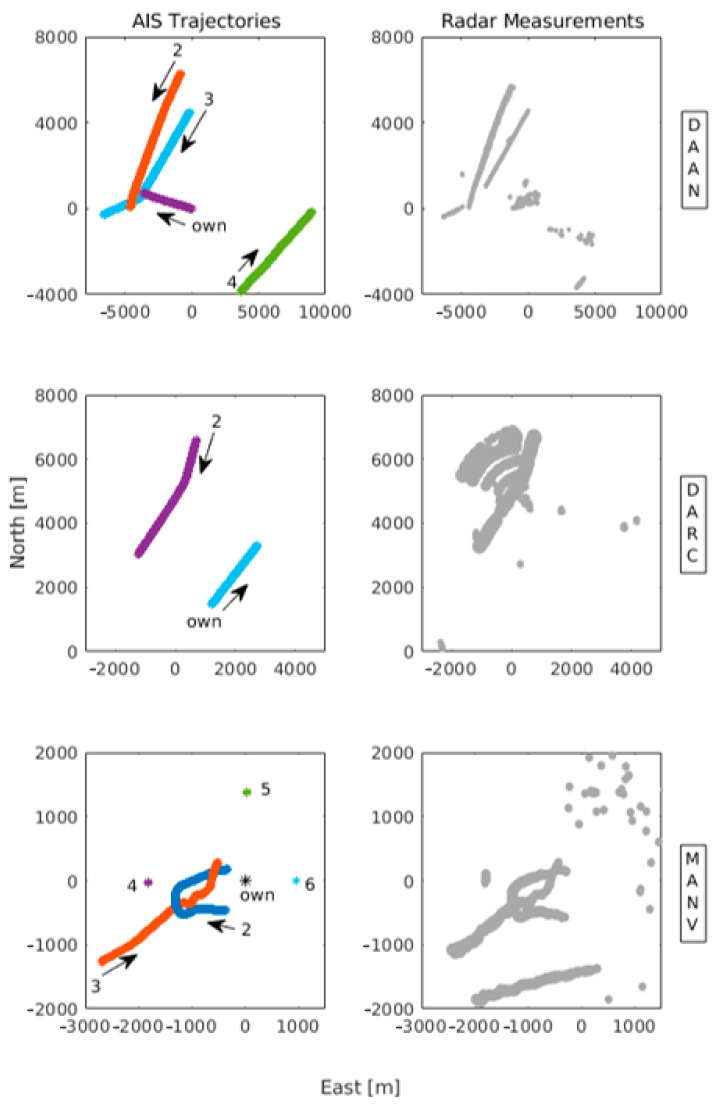
Depiction of the datasets’ trajectories. The left column shows the AIS-based reference trajectories of vessels for each dataset, all labelled. The arrows indicate the course of the vessels. The right column shows the radar point clouds measurements. Notice the trails of radar reflections in DAAN (*Data Association with Aids to Navigation*), the radar beacon (RACON)-originated measurements in DARC (*Data Association with RACON*) and the aids to navigation. In MANV (*Manoeuvres*), an unknown target has also been detected.

The DLR radar repository, under the own company’s export control principles, can be acquired as a zipped file (dataset-wise) from the following DOI: https://doi.org/10.26090/rwmr. It is to be noted that the datasets are open for requests from the EU and NATO member states; requests from other countries are subject to individual assessment by the approval board. The aim of using these selected trajectories from some measurement campaigns was to capture real-world situations as closely as possible on common navigation routes. Some basic details of each dataset, that is, the number of targets and frames captured, the dataset duration and the sensors involved, are summarised in Table 1. The dataset-specific trajectories (AIS and detected radar measurements) are illustrated in appropriate plots in Figure 2.

### 2.1. Campaign Setup 

In each campaign, either an own vessel or a participating vessel (as for MANV) was on course. Frame grabbers were used to capture the radar (X-band) screen visuals at a frequency of 1 Hz, during which some random data loss occurred as seen in Table 1. These images were further processed to automatically extract potential targets as point clouds by the method defined in Section 3. 

Selected AIS information that were recorded have been included, such as the position, dimensions, course over ground, speed over ground and true heading of the vessels equipped with transponders. Due to security and privacy related reasons, information divulging the identity of the vessels (from the unique maritime mobile service identification referred to as the MMSI), and the date and time of campaigns have been omitted. Furthermore, we note that the regions of interest have been more or less constrained to a coverage of their own vessels’ radar ranges, and that different (own) vessels were part of the specific campaigns. The dimensions of AIS-equipped targets involved are summarised in Table 2.

### 2.2. Additional Processing 

The initial AIS messages received were decoded and filtered based on the vessels’ MMSIs to eliminate unwanted data and outliers. The filtered information initially contained vessel positions in geodetic coordinates, that is, in latitude and longitude (where altitude was assumed negligible). In case more than one message were received at a specific time, the most recent message was considered. The positions were then converted to some local East North Up (ENU) coordinate system using an appropriate reference point location pivoting a tangent plane approximating the Earth’s surface. In general, AIS information that is transmitted is dependent on the speed of the vessel: higher speeds result in more frequent updates. The concerned AIS positional measurements were linearly interpolated to match the standard radar-grabber frequency of 1 Hz. For the datasets, a linear interpolator was enough to obtain the missing points. The AIS data can be found in *referenceMeas.mat*. 

As for the radar images, they have been processed by cropping out the user-interface functions so that only the region of interest is visible together with the sensor range values. This step was vital to remove real-world identifiable data such as the date and time of the experiment. The processed images are stored in the folder called *radarImages*.

The detected point clouds (obtained from the approach in the next section) are stored in the *polarMeas.json* and *cartesianMeas.json* files, both in the polar and Cartesian coordinates format. A sample code (*demo_visualise.py*) for visualizing the detections is also incorporated.

## 3. Target Detection and Tracking

**Figure 3:**
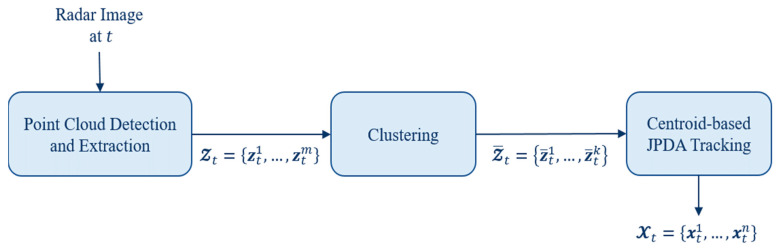
Illustration of blocks in the processing chain at observation step t.

In this section, we describe the methods used on the datasets from detecting to tracking the targets. Our overall processing chain at observation step t is shown in Figure 3, and the following subsections cover the methods and the appropriate data flow.

### 3.1. Automatic Target Detection

The captured radar images were each processed sequentially in chronological order and the positions of the potential targets stored in appropriate files were then fed to the baseline tracker. Steps followed for the image processing are as described:

(a) Each image is masked, hence eliminating further interface information (more specifically, the centre point, bearing line and the compass frame itself) and converted to grayscale.
(b) Using specific thresholds for intensity values, much of the weaker blobs have been filtered out. This reduces clutter from within the observation region.
(c) The blob detection algorithm, *Determinant of Hessians* (DoH) from Python’s *skimage* library (Blob Detection. Available online: https://scikit-image.org/docs/dev/auto_examples/features_detection/plot_blob.html (accessed on 2 July 2021)), is applied next for automatically detecting potential targets based on their distributions.
(d) The cloud of points (or measurements) that fall under the detected blob’s radius are extracted and stored in range (metres) and bearing (radians).

This method has been improved upon from the manual target versions in the previous works [22,23]. The DoH method provides an efficient scale- and rotation-invariant blob detection technique. A descriptor is represented by the determinant of convolved second-order Gaussian derivative (Hessian) elements that intuitively models the distribution of intensity within a region of interest [27]. 

On the basis of the AIS-retrieved own vessel’s positions, the point clouds’ coordinates have also been converted to a Cartesian ENU coordinate representation for both processing and visualisation simplicity [28]. At this point, the set of measurement $Z_{t}$ contains all the detected points of cardinality m. Note, for MANV, a pixel represented a finer resolution of 6 m while for the other two datasets, a pixel represented a coarser 11 m resolution.

### 3.2. Clustering

**Figure 4:**
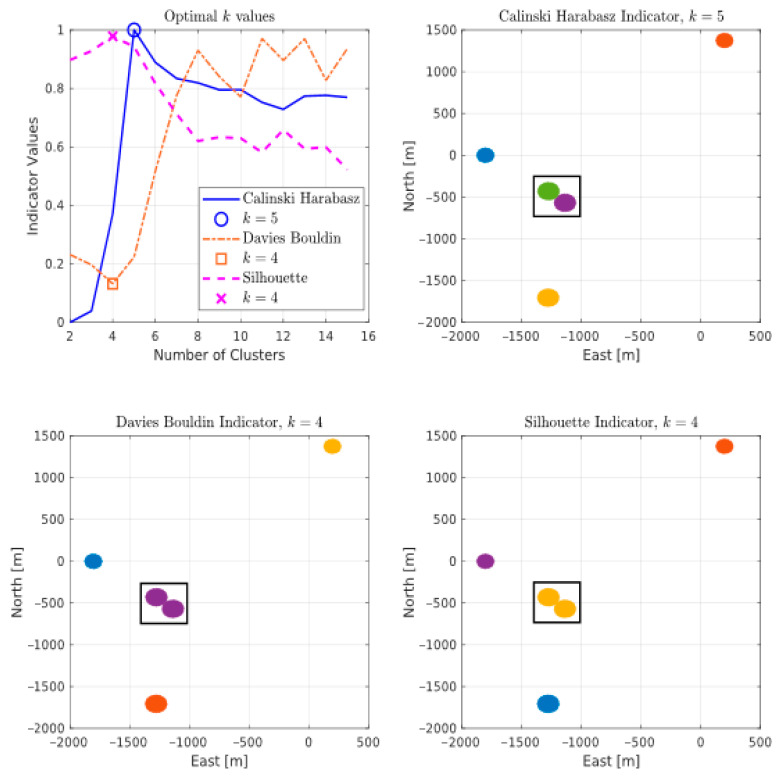
An exemplar output of cluster evaluation on MANV at observation step t=420 s. The upper left plot displays the criterion results denoting the optimal number of clusters k that were evaluated by the different indicators. The remaining plots are clustering results using the respective indicators. Calinski-Harabasz (**upper right**) was able to discern between point clouds from two targets while the remaining methods in the lower row, namely Davies Bouldin (**left**) and Silhouette (**right**), failed to. The indicator values for Calinski-Harabasz have been normalized for the sake of uniform comparison in the plot.

To calculate the centroids from the potential targets that have been extracted as point clouds, we perform k-means partitional clustering. While there are more advanced algorithms available for the data clustering [29,30], this method was chosen for its direct and simple approach. 

k-means is an iterative method that partitions data into k distinct subgroups (clusters) such that the Euclidean distance between a point and its assigned cluster’s centroid is at a minimum [24,25]. As the k value is normally predefined, we choose to instead autonomously find an optimal value for k by executing a cluster evaluation prior to the clustering. Figure 4 shows the results of three different evaluation and measure indicators for clustering [29,30] on an exemplar set of point clouds from MANV. In the case of our datasets, the Calinkski-Harabasz indicator was employed since it gave a more realistic clustering output. 

Given that a cluster consists of ℓ number of points, its centroid is calculated as $\bar{z}=\frac{1}{\ell }\sum_{i=1}^{\ell }z_{i}$. Thus, the set ${\bar{Z}}_{t}$ consists of the clusters’ centroids of cardinality k≪m that are then passed on as measurements to the filter described in the next parts.

### 3.3. EC-JPDA Tracker 

At observation step t, there is a multi-target state $X_{t}={\left\{x_{t}^{i}\right\}}_{i=1}^{n}$, that represents a set of n individual target states, is to be estimated from the centroid measurement set ${\bar{Z}}_{t}={\left\{{\bar{z}}_{t}^{j}\right\}}_{j=1}^{k}$.

The JPDA method, which associates validated measurements to targets based on their specific probabilistic weights, is employed. An extended Kalman filter (EKF) [31] is then used for the state and state covariance estimations.

#### 3.3.1. Dynamic and Measurement Models

A single target state $x_{t}={\left[p_{\tilde{e},t},p_{\tilde{n},t},\;\varphi_{t},\vartheta_{t}\right]}^{T}$ contains the kinematic parameters of interest such as the vessel position $\left(p_{\tilde{e}},p_{\tilde{n}}\right)$ in local ENU coordinates, its respective course  φ and speed ϑ over ground, where T is the transpose operator. Target motion is modelled by a non-linear function described as:

(1) $$\begin{array}{cc} x_{t} & =g\left(x_{t-1},\;u_{t}\right)\\ & =\left[\begin{array}{c} \begin{array}{c} p_{\tilde{e},t-1}+\;\sin(\varphi_{t})\cdot dt\cdot \vartheta_{t}\\ p_{\tilde{n},t-1\;}+\cos(\varphi_{t})\cdot dt\cdot \vartheta_{t} \end{array}\\ \varphi_{t}\\ \vartheta_{t} \end{array}\right]+u_{t} \end{array}$$

where $u_{t}\sim N\left(0,Q\right)$ is a zero-mean process noise with covariance Q and dt is the time interval in-between observation steps. The measurement equation relating a measurement to some target origin is:

(2) $$z_{t}=Hx_{t}+v_{t}$$

with an additive zero-mean measurement noise $v_{t}\sim N\left(0,R_{t}^{j}\right)$ and measurement matrix $H=\left[\begin{array}{cccc} 1 & 0 & 0 & 0\\ 0 & 1 & 0 & 0 \end{array}\right]$. $R_{t}^{j}$ is the measurement noise covariance and is calculated based on the conversion formulae presented in [28].

#### 3.3.2. Gating

For an efficient measurement processing, multiple stages of gating were carried out to eliminate measurements away from predicted target position: a rectangular one for ${\bar{Z}}_{t}$ and an ellipsoidal gating [20,26]. The rectangular gating is applied to the centroid measurements in ${\bar{Z}}_{t}$ at first. The ellipsoidal gating is then applied again to the centroid(s) selected from the rectangular gate, including the measurements in $Z_{t}$ that belong to the validated centroid’s cluster points. The region enclosed as a consequence of ellipsoidal gating, also known as validation region $G_{t}^{i}$, is denoted by:

(3) $$G_{t}^{i}\;=\left\{z:\;{\left(\bar{z}-{\hat{z}}_{t|t-1}^{i}\right)}^{T}{\left(S_{t}^{i}\right)}^{-1}\left(\bar{z}-{\hat{z}}_{t|t-1}^{i}\right)<\gamma \right\}\;$$

where ${\hat{z}}_{t|t-1}^{i}$ is the predicted position measurement of target i, and γ is the gate threshold determined by some given gating probability. $S_{t}^{i}$ is the innovation covariance calculated as follows:

(4) $$S_{t}^{i}=HP_{t}^{i}H^{T}+R_{t}^{j}+{\hat{S}}_{D,\;t-1}^{i}\;$$

**Figure 5:**
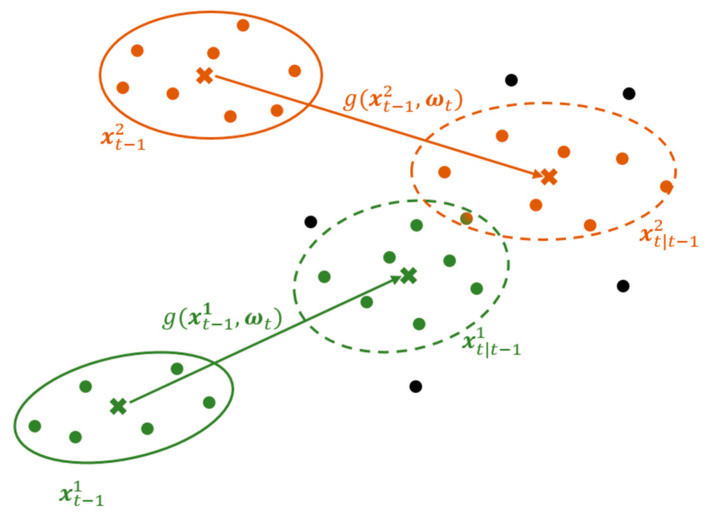
Visualisation of measurement validation using gating method calculated as in (3)–(5). Measurements that fall within validation regions of the two respective targets are used to calculate the dispersion matrix for the next observation step. Unvalidated ones are neglected while common ones are shared.

$P_{t}^{i}$ is the covariance matrix of target state $x_{t}^{i}$. We define ${\hat{S}}_{D,\;t-1}^{i}$ as an adaptive dispersion matrix that takes into consideration the spread of the measurements from the immediate previous step, assumed to have originated from the target’s body and that fall within the validation region. This can be visualised as shown in Figure 5 and is given by:

(5) $${\hat{S}}_{D}^{i}\;=\;\left[\begin{array}{cc} \sigma_{\tilde{e}}^{2} & \sigma_{\tilde{e}\tilde{n}}^{2}\\ \sigma_{\tilde{e}\tilde{n}}^{2} & \sigma_{\tilde{n}}^{2} \end{array}\right]\;$$

with $\sigma_{\tilde{e}}^{2}$ and $\sigma_{\tilde{n}}^{2}$ being the variances of the validated measurements in the East and North directions and $\sigma_{\tilde{e}\tilde{n}}^{2}$ represents the cross-covariance in the East and North directions.

#### 3.3.3. JPDA Filter

In this part, we briefly go over the main steps for calculating the joint association probabilities of centroid measurements to targets. More elaborate explanations of the same can be found in the basic works [8,14,19]. 

At first, a binary validation matrix Ω, of size k×(n+1), is generated from $G_{t}^{i}$ such that all feasible centroid-target pairings are represented as:

(6) $$Ω=\;\left[\omega_{ji}\right]\;$$

such that:

(7) $$\omega_{ji}=\;\{\begin{array}{cc} 1 & \mathrm{if}\;\mathrm{centroid}\;j\;\mathrm{is}\;\mathrm{in}\;G_{t}^{i}\;\\ 0 & \mathrm{otherwise}. \end{array}$$

Under the 1-1 mapping constraint between a centroid and a target, the association matrix $\hat{Ω}$ comprising of joint events is generated. These are all the possible individual events χ  whereby a centroid j could be associated with a single target except for i=0, which signifies association with clutter instead:

(8) $$\hat{Ω}\left(\chi \right)=\left[{\hat{\omega }}_{ji}\left(\chi \right)\right]\;$$

${\hat{\omega }}_{ji}=1$ if and only if the hypothesis $\chi_{ji}$ that centroid j is associated either to a target i (0<i≥n) or to clutter otherwise. The joint association probabilities of event $\chi \left(\hat{Ω}\right)$ are calculated based on:

(9) $$P\left\{\chi \left(\hat{Ω}\right)|{\bar{Z}}_{t}\right\}=\;\frac{1}{c}\;\underset{j:\;\omega_{ji}=1}{\prod }\lambda^{-1}P\left({\bar{z}}_{t}^{j}|\chi_{ji},{\bar{Z}}_{t-1}\right)P\{\chi \left(\hat{Ω}\right)|k,\;{\bar{Z}}_{t-1}\}$$

where c is a normalising constant summing the conditional probabilities $P\left\{\chi \left(\hat{Ω}\right)|{\bar{Z}}_{t}\right\}$ over all events $\chi \left(\hat{Ω}\right)$ and λ the clutter density over the observation region. A validated centroid measurement ${\bar{z}}_{t}^{j}$ has a Gaussian density given by:

(10) $$P\left({\bar{z}}_{t}^{j}|\chi_{ji},{\bar{Z}}_{t-1}\right)=\;\{\begin{array}{cc} N\left({\bar{z}}_{t}^{j};\;{\hat{z}}_{t|t-1}^{i},\;S_{t}^{i}\right) & \mathrm{if}\;\omega_{ji}=1\\ V^{-1} & \mathrm{otherwise}. \end{array}$$

V is the volume of the validation region while ${\hat{z}}_{t|t-1}^{i}$ is the predicted position and $S_{t}^{i}$ is the innovation covariance as calculated from (4). The prior probability, that is, the last factor in Equation (9) is:

(11) $$P\left(\chi \left(\hat{Ω}\right)|k,\;{\bar{Z}}_{t-1}\right)=\;\underset{i}{\prod }P_{D}^{\delta_{i}}\;{\left(1-P_{D}\right)}^{1-\delta_{i}}$$

where $\delta_{i}$ is the target association indicator such that $\delta_{i}=\;\sum_{j=1}^{k}{\hat{\omega }}_{ji}\left(\chi \right)$.

The marginal association probability $\beta_{ji}$ the jth centroid measurement is associated with target i. is now found by summing over all feasible events $P\left\{\chi \left(\hat{Ω}\right)|{\bar{Z}}_{t}\right\}$ using:

(12) $$\beta_{ji}\;≜\;\underset{\chi \left(\hat{Ω}\right)}{\sum }\;P\left\{\chi \left(\hat{Ω}\right)|{\bar{Z}}_{t}\right\}{\hat{\omega }}_{ji}\;$$

These values are then used as weights for state estimation of each track from the standard EKF equations [31].

### 3.4. Track Initiation and Estimate Evaluation 

In this work, the tracks have been initialised from some initial guesses around the first AIS reference positions of the tracks wherever available (qualified tracks) and otherwise around the radar measurements (as it is in the case of the AIS transponder-unequipped craft in MANV). The Euclidean distance-based positional errors are used to evaluate the filter estimates against the reference. 

## 4. Results

In this section, we demonstrate the tracking results and carry out some analysis on all three datasets. Two versions of JPDA are considered. The first one is the standard JPDA filter as conceived in [20], and the second one is our proposed approach, EC-JPDA, which is an adapted version of the former. 

**Figure 6:**
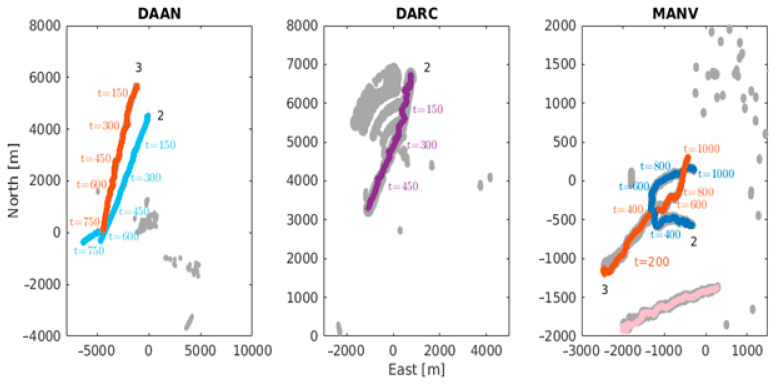
The estimated trajectories (coloured) and the radar point cloud measurements (grey dots) are shown following matching annotation and colour scheme from Figure 2 as convention. The observation steps are also printed for additional reference in the discussion.

**Figure 7:**
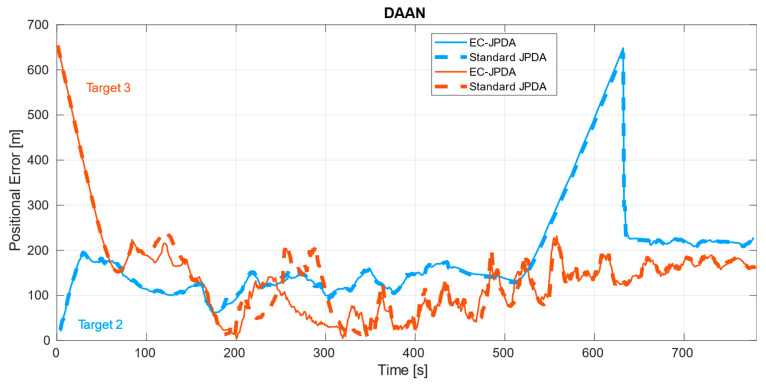
The positional error of the two targets in DAAN.

**Figure 8:**
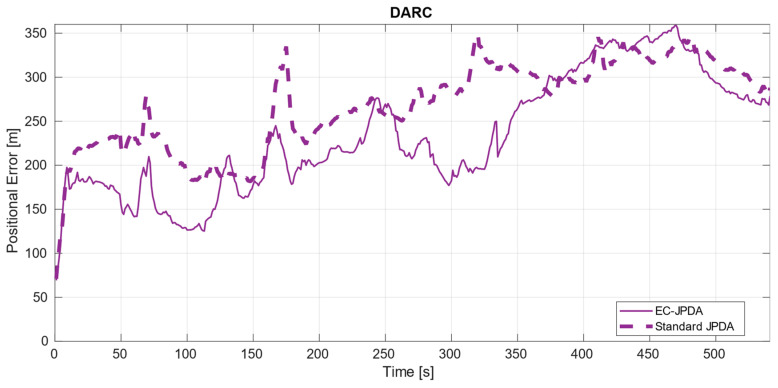
Error of Target 2, a large vessel, in DARC.

**Figure 9:**
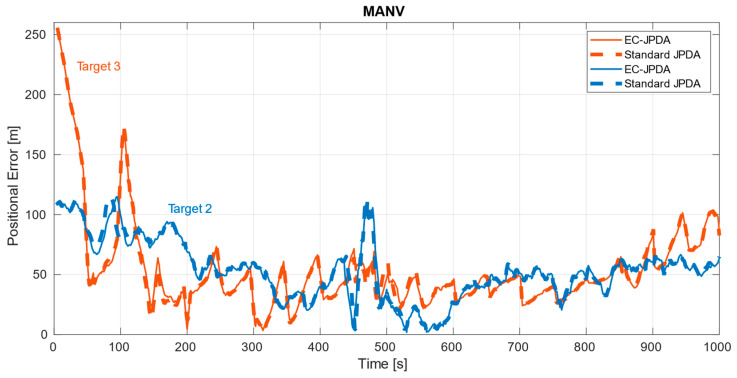
Errors of the two qualified targets in MANV.

While they both consider the centroids as measurements, the basic difference between the two filters is in the gating and accounting the dispersion matrices of the centroids considered for the adapted version, as explained in the previous section. Figure 6 shows the output of our tracker. The estimates have been plotted on top of the radar measurements and the observation steps are included to facilitate the discussion ahead. The error plots are depicted in Figure 7, Figure 8 and Figure 9 in a dataset-wise order for the qualified tracks. In the discussion, the errors are explained more in details with respect to the trajectories before comparing the performance of the EC-JPDA version to the standard JPDA version. 

### 4.1. Discussion

The estimated tracks for DAAN show continuous and properly associated measurement to track, in particular around the point of closest approach of Target 2 to the aid to navigation which is around t=200. Due to a series of missed detections because of occlusion between approximately t=500 to t=630 the estimated trajectories relied more on the process model before getting back on track. The error values are higher for Target 3 at the beginning due to its poor and limited visibility via the radar sensor. 

In the case of DARC, despite having the RACON-originated measurements often interfering with those of the true target, the track was well estimated as seen in Figure 6. The error peaks up to t=400 correspond to the RACON’s and aids to navigation interferences throughout the trajectory. This is due to the clustering process being affected that naturally make centroid values quite inaccurate. From t=400 onwards, there are two combined reasons to explain the increase. First is the presence of another aid to navigation and second, the own sensor’s perspective over the vessel changing—which causes the AIS measurements to have a lead over the radar measurements.

A note here is to also consider the dimensions (129 × 23 m and 180 × 28 m) of the large targets being tracked in DAAN and DARC, and the fact that the AIS antenna might not ideally indicate the centre of the vessel. Detections that come from over the target’s surface can cause significant jumps in the centroids that end up affecting the estimations as well.

As for MANV, the tracks were also clear in addition to an unreported AIS track (moving from left to right denoted in pink), that was most likely from a leisure craft not equipped with any AIS transponder. The dataset has a relatively finer resolution and smaller vessels, hence the overall error in comparison to the other two datasets is lower. There are however more fluctuations here because of the targets’ manoeuvres and brisk speed and course changes throughout the whole duration. The peak between t=400 to t=500 occurs at the targets’ intersection region, where the measurements associations were affected. The high error at the beginning for Target 3 corresponds mainly to its poor visibility. Around the end, it was partially occluded by Target 2.

### 4.2. Comparison to Standard JPDA

While the errors of the two filter versions for the targets in MANV and Target 2 in DAAN were almost overlapping, the errors in DARC and Target 3 in DAAN were disparate. There is a higher improvement particularly in DARC throughout the entire trajectory, the most challenging dataset considered. For Target 3 in DAAN, the improvement occurs during the first half where the visibility is rather poor, with occasional moments of occlusion. As soon as visibility enhances, the errors overlap. The dispersion matrix in EC-JPDA takes the measurements distribution into account thereby making the filter more robust than its counterpart in the noisier surroundings by retaining information related to the target’s underlying body. This also implies that there is also a relevant correlation of the range of the own vessel with respect to the target to the estimate’s accuracy: closer and undisturbed measurements from close ranges offer similar results for both versions, in contrast to the distant and highly noisy measurements obtained from farther ranges. In the latter case, the EC-JPDA filter could provide more accurate results.

### 4.3. Limitations

The onboard marine radars have limited sensor resolution, which when considered together with the typical radar imperfections (smearing and reflections for instance) make solving applications such as ETT more challenging. In general, the precision is decreased for both the kinematics and the perceived size of the vessel, which somehow distorts the values of the dispersion matrix and leads to inaccuracies during tracking. 

Furthermore, the tracking results are heavily dependent on the clustering results and tend to vary between runs. As k-means was used, the drawbacks of the method are also applicable in our case—it does not suit different densities but instead shows an affinity towards spherical clusters. The elongated point clouds, for instance, could be detected as at least two clusters instead of one.

## 5. Conclusions

In this article, we have presented the DLR image-based marine radar datasets—that are available to the community for research purposes. The datasets aim at advancing the state of the arts methods in particularly maritime research, as they capture very maritime route-specific challenges such as aids to navigations and RACONs.

Altogether, we also present a blockchain to explain our target detection, measurement clustering and MTT approaches respectively. As some first step, a new adaptation for MTT that basically uses a centroid-based JPDA, called EC-JPDA, was proposed as a baseline algorithm. The results of EC-JPDA tracker and a standard JPDA tracker run on the dataset was shown and the results were discussed. 

Overall, it was deduced that EC-JPDA outperformed the standard one on a very noisy and challenging dataset. The performance was similar to the standard one under some more ideal conditions (less noise, close-range). 

## Figures and Tables

**Table 1:** Summary of the datasets.

Dataset	# Targets	# Frames	Duration [s]	Onboard Radar Sensor(s)
DAAN	4	780	780	Dynamic (own)
DARC	2	527	540	Dynamic (own)
MANV	6	976	1000	Static (observer)
		1000	1000	Dynamic (own)

**Table 2:** Dimensions of vessels for the different datasets.

Dataset	Participating Targets	Length [m]	Width [m]
DAAN	Target 2	129	23
	Target 3	12	4
DARC	Target 2	180	28
MANV	Target 2	29	7
	Target 3	23	6

## Data Availability

The data that support the findings of this study are available at https://doi.org/10.26090/rwmr (accessed on 2 July 2021) under DLR’s export control principles.
